# Evolution of Lightning Maculopathy: Presentation of Two Clinical Cases and Brief Review of the Literature

**DOI:** 10.1155/2021/8831987

**Published:** 2021-01-22

**Authors:** Simanta Khadka, Raghunandan Byanju, Sangita Pradhan, Suchan Poon, Rinkal Suwal

**Affiliations:** ^1^Bharatpur Eye Hospital, Bharatpur, Chitwan, Nepal; ^2^BP Eye Foundation, Hospital for Children, Eye, ENT, and Rehabilitation Services (CHEERS), Madhyapur Thimi, Bhaktapur, Nepal

## Abstract

Lightning is a naturally occurring atmospheric phenomenon. Though uncommon, it is a potentially devastating and underreported natural calamity. Lightning accounts for the second leading cause of weather-related death in most parts of the world. Among the survivors of lightning injury, more than half of the victims may suffer from some form of ophthalmic injury. The lightning-associated ocular injury varies from a range of anterior segment to posterior segment pathologies. We report on two clinical cases of ocular injuries among the survivors of lightning injury. Anatomical involvement is seen at different levels with presentation as uveitis, pupillary abnormality, maculopathy, and later development of lenticular opacification. Optical coherence tomography (OCT), a noninvasive diagnostic tool, is particularly useful in the evaluation of lighting maculopathy as well as to monitor its progression through the course of time. Visual prognosis depends upon the structures of the eyes affected in the injury. The presence of irreversible retinal damage as well as optic nerve damage often result in poor visual outcome in the absence of significant anterior segment pathology. This report highlights the evolution of maculopathy through the course of time and signifies the importance of long-term follow-up postlightning injury.

## 1. Introduction

Lightning is a naturally occurring atmospheric phenomenon. It is estimated that the earth is hit by lightning more than 100 times in each second, and around 50,000 thunderstorms occur every day, resulting in fire, injury, and even loss of life or properties [1]. The odds of being struck by lightning are about 1 in 960,000; however, almost 9 out of 10 victims may escape death [2]. Electrocution or electrofulguration is the commonest mode of injuries due to lightning strike accounting for high morbidity (75%) and mortality (20-30%) [3–5]. The most common cause of death is attributed to ventricular fibrillation or asystolic cardiac arrest [6].

Among the survivors of lightning injury, more than half of the victims suffer from some form of ophthalmic injury [7]. Numerous literatures have reported the spectra of lightning strike-associated ocular injuries [8–18]. The ocular injuries can vary with a range of anterior segment pathologies [16], consisting of thermal keratopathy, hyphema, uveitis, and cataract to posterior segment pathologies, which constitute of vitreous and retinal hemorrhage, cystoid macular edema, retinal detachment, retinal vascular occlusion, chorioretinal rupture, macular hole, and lightning maculopathy [9, 16, 19]. Neurological injuries, namely, optic neuropathy, papillitis, anisocoria, cranial nerve palsies, nystagmus, abnormal pupillary reflex, and Horner syndrome have also been reported in few instances [16, 19].

Here, we report on two cases of ocular injuries among the survivors of lightning injury. Anatomical involvement is seen at different levels with presentation as uveitis, pupillary abnormality, maculopathy, and later development of lenticular opacification. We describe the evolution of maculopathy demonstrated by optical coherence tomography (OCT) and include a brief review of the literature.

## 2. Case Description

### 2.1. Case 1

**Figure 1:**
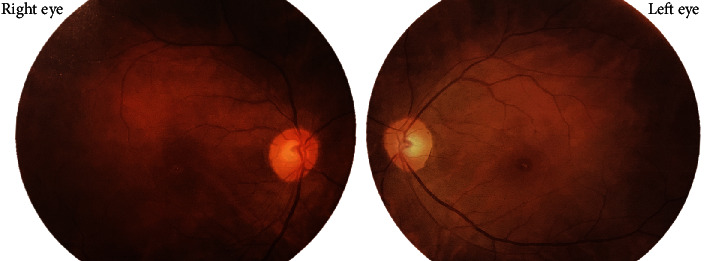
Fundus photograph at the time of presentation. Presence of bilateral dull foveal reflex and central yellow-white lesion surrounded by orange-brown pigmentation is shown in the macula. The lesion is prominent in the left eye.

**Figure 2:**
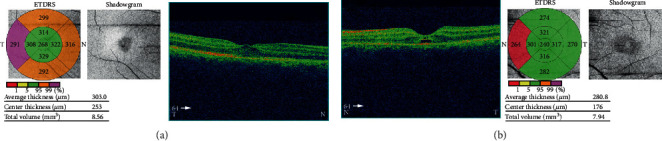
Spectral-domain optical coherence tomography (SD-OCT) at presentation. (a) Right eye with few intraretinal cystoid changes. (b) Left eye with a foveal cyst.

A 37-year-old male with unremarkable medical history presented with bilateral diminution of vision. The symptoms began three days prior to presentation when he was sleeping on a floor of a rural house (cemented floor, brick walls, and galvanized zinc roof). He recounted being woken briefly by a sudden appearance of a bright flash from a nearby lightning (approximately six meters away) during the rainy season of July 2018, following which he had loss of consciousness lasting for approximately 45 minutes. After regaining consciousness, he noticed blurring of vision in both eyes (BE), which improved initially but worsened afterward for which he sought ophthalmic consultation. He also noticed decreased hearing in the left side. On initial evaluation, his best-corrected visual acuity (BCVA) was 6/18 in the right eye (RE) and 6/60 in the left eye (LE). His ocular movement was preserved. The eyelids, ocular adnexa, and pupils were symmetrical, but there was relative afferent pupillary defect in LE. The rest of his anterior segment evaluation was within normal limits including absence of lenticular opacification but there was presence of 1+ anterior chamber (AC) cells in LE. Dilated fundus evaluation revealed a bilateral dull foveal reflex simulating a macular hole- (MH-) like appearance in BE (Figure 1). However, the Watzke-Allen test result was negative. The intraocular pressure (IOP) was 14 mmHg in RE and 16 mmHg in LE. Spectral-domain optical coherence tomography (SD-OCT) (Topcon 3D OCT, 2000 series, Japan) demonstrated evidence of intraretinal cystoid abnormalities prominent in RE, subfoveal outer retinal hyporeflective space suggestive of foveal cyst in LE, and increased retinal pigment epithelium (RPE) hypereflectivity in BE (Figure 2). He was treated with topical steroid and cycloplegic agent in BE.

**Figure 3:**
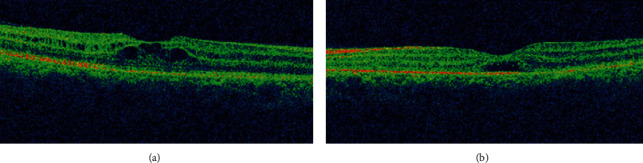
SD-OCT at four weeks following lightning injury. (a) Right eye with intraretinal cystoid changes. (b) Left eye with a foveal cyst. The cyst has increased fluid volume compared to OCT at presentation.

He then presented four weeks later with slight improvement in BCVA of 6/12 in RE, but the vision in LE was static. The SD-OCT showed intraretinal cystoid changes in the middle retinal layers which was exaggerated compared to previous scan in RE (Figure 3(a)), and persistent foveal cyst appeared as hyporeflective lucency in the outer retinal layer just anterior to retinal pigment epithelium in LE (Figure 3(b)). He was then continued on topical steroid and cycloplegic agent for four more weeks.

**Figure 4:**
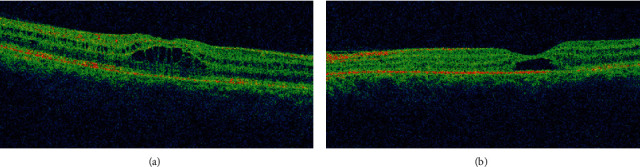
SD-OCT at two months following injury. (a) Right eye with interval development of intraretinal cystoid abnormalities. (b) Left eye with persistent foveal cyst.

After two months of injury, his BCVA improved to 6/9 in RE only and 6/60 in LE. Fundus examination revealed an interval development of central macular thickening, and the SD-OCT confirmed intraretinal cystoid changes with disruption of the subfoveal ellipsoid zone in RE. Similarly, the foveal cyst was continual in LE (Figure 4). He was then prescribed topical nonsteroidal anti-inflammatory drugs in BE. He pursued his profession as a cook overseas and committed to seek medical advice in case of need.

**Figure 5:**
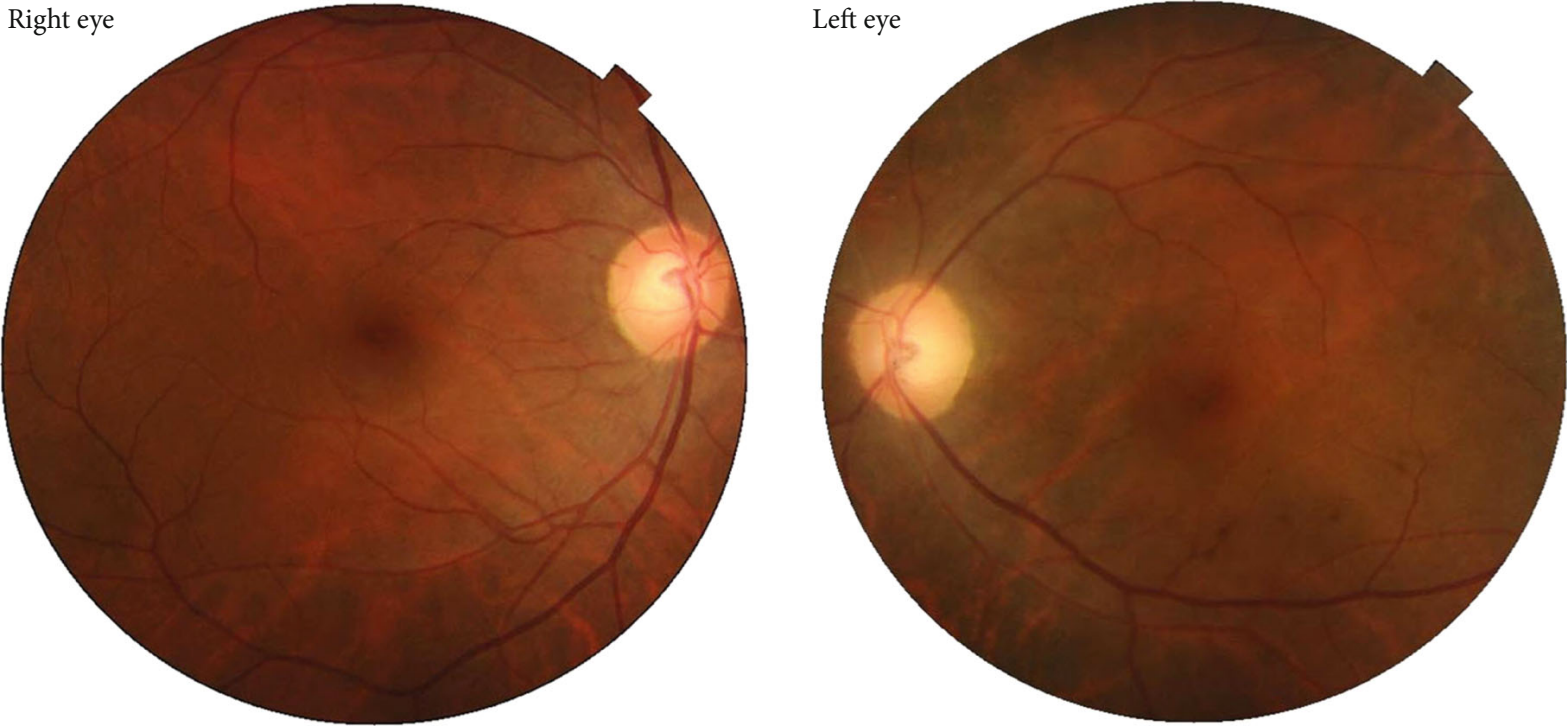
Fundus photo at the end of one and half-year following the injury.

**Figure 6:**
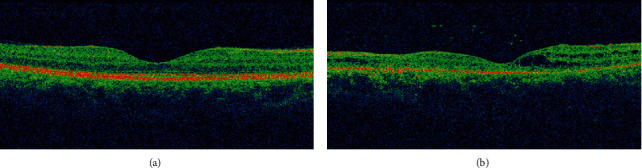
SD-OCT at one and half-year follow-up. (a) Right eye with near normal restoration of foveal anatomy. (b) Left eye showing few cystoid abnormalities but atrophy of the nasal retina.

He followed up with us at an interval of one and half-year following the injury upon his arrival. The documented BCVA was 6/9 RE and 6/60 LE. A mild cortical and posterior subcapsular cataract was evident on slit-lamp examination. Fundus examination showed dull foveal reflex (Figure 5). The Watzke-Allen test was negative, but he still complained of mild distortion in the Amsler grid test of LE. OCT affirmed the restoration of foveal contour in RE (Figure 6(a)). Nonetheless, there was still presence of few intraretinal cystoid abnormalities in LE. The previous hyporeflective foveal cyst was absent, and further loss of the outer retinal layers was evident in the macula (Figure 6(b)).

**Figure 7:**
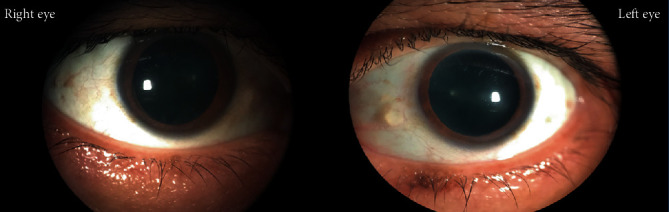
Anterior segment diffuse photography at the end of two years follow-up. Anterior subcapsular opacification is visible in the photography, which is more prominent in the right eye compared to the left.

**Figure 8:**
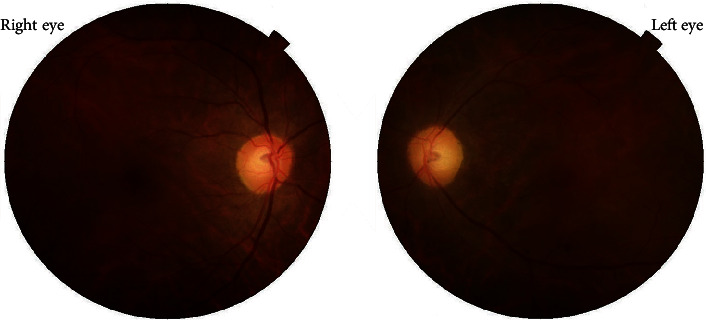
Fundus photo at the end of two years follow-up showing dull foveal reflex in both eyes.

**Figure 9:**
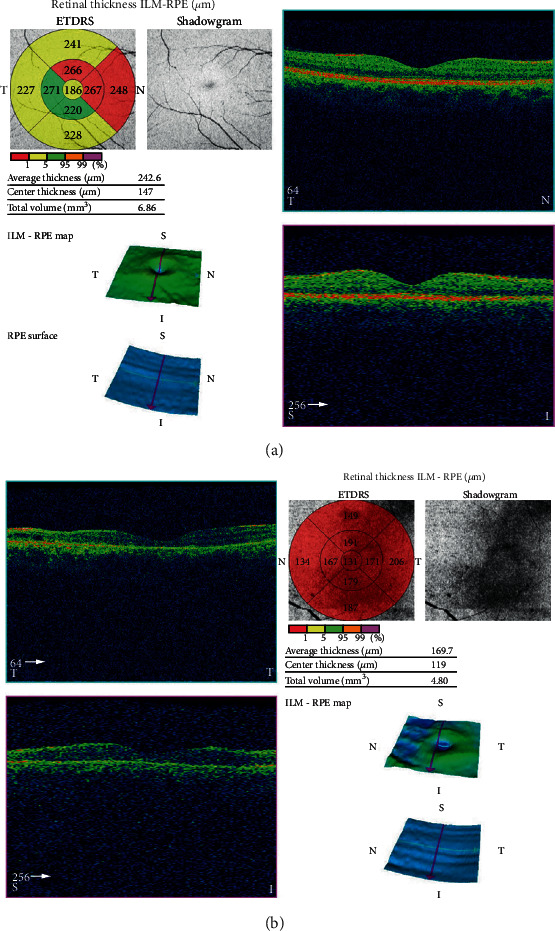
OCT at the end of two years follow-up. (a) Right eye with restoration of foveal anatomy. (b) Left eye with gross retinal atrophy.

His continual follow-up with us at the passage of two years postlightning injury (July 2020) revealed a BCVA of 6/9 RE and 6/60 LE. Early anterior and posterior subcapsular lenticular opacification was present during slit-lamp examination (Figure 7) and a dull foveal reflex in dilated fundus examination (Figure 8). Automated Humphrey 24–2 Swedish Interactive Threshold Algorithm (SITA) standard visual field analysis revealed mild deficit extending from the blind spot in the left eye. OCT revealed normal restoration of foveal anatomy in RE (Figure 9(a)), whereas the intraretinal cystoid abnormality was almost absent but the nasal retina was grossly atrophic in LE (Figure 9(b)).

### 2.2. Case 2

**Figure 10:**
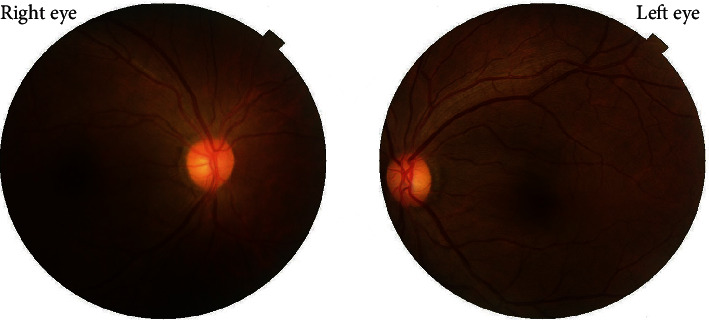
Fundus photography at presentation showing macular lesion simulating macular hole. Bilateral central yellow-white lesion surrounded by orange-brown pigmentation in the macular region is visualized.

**Figure 11:**
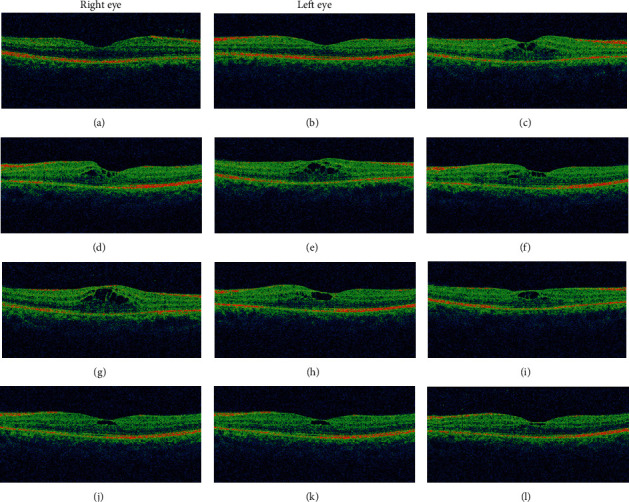
(a)–(l) Serial OCT scans showing the evolution of maculopathy obtained at the time of presentation to the last follow-up visit at the end of five months postinjury. (a, b) OCT at presentation showing normal appearance. (c, d) OCT at three weeks of injury with development of intraretinal cystoid abnormalities. (e, f) Five weeks following injury showing increment in cystoid changes. (g, h) Interval increment in macular thickness as well as cystoid pockets more marked in the right at the end of two months. (i, j) Four months interval with decreased cystoid changes in both eyes. (k, l) OCT at the end of last follow-up visit at five months showing resolution of cystoid spaces and restoration of fovea in the right; however, presence of foveal cyst in the left was evident.

A 42-year-old male with no ocular and medical history was struck by lightning (June 2019) while on his way back home resulting in a loss of consciousness. Upon arousal after 20 minutes, he complained of pain and swelling of the right side of his face. He was then brought to the emergency department of a general hospital where the primary management of his condition was done, and the neuroimaging revealed no abnormality. He was then referred to us for the complaint of BE blurring of vision associated with pain and photophobia after two days. Upon evaluation, the BCVA was 6/24 in RE and 6/18 in LE. The ocular motility was full in all cardinal gazes, and no pupillary abnormality was noted bilaterally. Anterior segment examination showed mild conjunctival congestion in BE with presence of 2+ AC cells in RE and 1+ AC cells in LE. Dilated fundus examination revealed bilateral dull foveal reflex and bilateral central yellow-white lesion surrounded by orange-brown pigmentation in the macular region (Figure 10). SD-OCT did not divulge any significant abnormality except for slight blunting of inner retinal layers more prominent in right (Figures 11(a) and 11(b)). He was treated with topical steroid and cycloplegic agent for traumatic iritis.

Three weeks after the injury, the BCVA was similar to the vision at presentation, but the patient complained of marked distortion. Upon examination, the iritis has quietened. The Watzke-Allen test was negative, but the distortion of the lines in Amsler grid was more pronounced. OCT showed an increase in central macular thickness with loss of foveal contour, intraretinal cystoid abnormalities in the middle retinal layers, and disruption of ellipsoid zone. The changes were more marked in RE compared to LE (Figures 11(c) and 11(d)). The patient was continued on topical steroid. On follow-up evaluation at five weeks postinjury, the BCVA had declined to 6/60 RE and 6/24 LE. Fundus examination showed interval development of macular edema BE, similarly OCT confirmed the increment in cystoid abnormalities more in the right (Figures 11(e) and 11(f)).

At the end of two months, the BCVA was restored as of baseline to 6/24 in RE, whereas it improved to 6/12 in LE. The OCT of RE was analogous to the previous finding, but increment in central macular thickness was noted. There was slight restoration of foveal anatomy but still presence of subfoveal cystic changes in LE (Figures 11(g) and 11(h)). He was advised to for topical nonsteroidal anti-inflammatory drugs BE. Serial OCT scans were obtained during his follow-up over the next two months. By the end of four months, the BCVA in RE was 6/24 and 6/12 in LE. Development of early posterior subcapsular cataract was evident during slit lamp examination. OCT demonstrated decreased intraretinal cystic changes in BE (Figures 11(i) and 11(j)).

Five months after the event, the BCVA RE improved to 6/18 RE and was static to 6/12 LE. Dilated fundus examination revealed no other findings except for dull foveal reflex. Repeated OCT scan established interval restoration of foveal anatomy in RE. The outer layers of the retina could be distinguished, and there was resolution of intraretinal cystic abnormalities, but the foveal contour was not restored completely (Figure 11(k)). Similarly, in LE, there was persistent cystic space with restoration of outer layers of the retina (Figure 11(l)). The patient lost for follow-up afterward.

## 3. Discussion

The patients discussed in our report developed maculopathy induced by lightning injury, which resembled MH in clinical appearance. This simulation is due to macular edema and cystic changes in the retina [9]. To the best of our knowledge, this could be the first case to report the evolution and sequelae of macular changes associated with lightning during a period of two years. Similarly, the data of the second patient was available for only up to five months following the injury, and then we lost the follow-up. In both cases, the documented anterior segment pathology of cataract was evident, and there was gradual progression in the opacification. Afferent pupillary defect was elicited from the time of presentation and persisted until the last follow-up in the first case. The injuries reported in our patients signify a multitude of ocular pathologies in lightning-induced injury to the eye.

Lightning accounts for the second leading cause of weather-related death in most parts of the world. Though uncommon, it is a potentially devastating and under reported natural calamity as it does not cause a mass casualty [20]. The distribution and frequency of lightning vary on different geographical locations and meteorological distribution across the globe [21]. Nepal, the Himalayan nation, is very much more prone to lightning activity which is favored by the surface temperature, atmospheric moisture, elevation, and the topography of this region [22].

The first documented report of lightning-induced ocular injury dates to 1722 by St Yves [23]. The possible mechanism of injury to the tissues by lightning may occur by several ways: direct strike, side flash or spray, or ground strike [16]. Direct strike occurs when the victim comes in direct contact with lightning, which is believed to be the most damaging mode of injury. The transmission can occur by touching the direct path of the current such as a pole or tree. Side splash, side flash, or spray injury can result when lightning jumps from the primary contact point to the victim [24]. If the lightning impacts the ground first and then spreads to a victim, a ground strike or stride injury can occur [25]. The injury reported in both of our patients is due to the ground strike current. The affection of hearing and ocular injury in the first case also supports the evidence of ground strike [8].

The power of lightning is estimated to be between 10,000 and 200,000 amperes of current, with an estimated voltage ranging from 20 million to 1 billion volts [26]. The extent of tissue destruction depends upon several variables, including the voltage, amperage, resistance, pathway, and duration of current flow [27]. When the eye is considered as a component of an electric circuit, the melanin granules of the RPE [28] and the uvea act as a main obstacle for the flow of the current [9]. The mechanism for tissue damage following lightning injury is attributed to direct electrolysis, resistance-induced heat, or mechanical disruption by the associated shock wave [11]. Secondary damage may also result from tissue edema, ischemia, or reperfusion injury [16].

The high melanin content of macula makes it highly sensitive to thermal damage. The resistance offered by melanin within the pigment epithelium of retina leads to heat production. Additionally, cylindrical shockwave produced by lightning can also lead to mechanical injury of the RPE. Localized inflammation could contribute to RPE dysfunction and retinal vascular incompetence resulting in decreased fluid transport across the retina and development of intraretinal edema [9]. The initial macular lesion with resemblance to Berlin's edema seen early after the lightning strike may be replaced or evolve into lesion later described as a intraretinal cyst or lesion simulating MH [19]. Negative Watzke-Allen test results, lack of posterior vitreous detachment, and operculum support the diagnosis of lightning maculopathy, which is a predominant feature of full-thickness MH [9, 14]. Macular cystic changes and full-thickness MH due to lightning need to be differentiated in the early stage as the management approach in this condition is completely different. Maculopathy with cystic change may spontaneously resolve with observation only, but for full-thickness MH, surgical intervention may be required [14]. However, lightning-induced MH may undergo spontaneous closure in a few instances [19]. OCT is helpful for delineating the difference in identification of MH and lightning-induced maculopathy. [14] The OCT appearance of the macular lesion shares a common appearance with solar retinopathy, [29] and welder's maculopathy [28] among other pathologies of photic maculopathy which is essentially due to a photochemical reaction. Hence, meticulous inquiry of the previous exposure of these factors needs to be excluded.

We were able to document the evolution of macular changes with the help of OCT. The change of macular lesions over the period of time also helps in predicting the prognosis [30, 31]. Apart from the solitary cystic changes in the macula previously reported through OCT, [32] we were able to demonstrate the delayed onset intraretinal cystic changes [33]. The eventual atrophy of the fovea due to obliteration of the nasal photoreceptor layer has led to deterioration of vision in the first case, and these changes are persistent with the previous report on evolution of lightning maculopathy [14, 31].

The inflammatory changes in the uveal tissues are also of frequent occurrence [15]. The iritis might appear within days to weeks following injury, but these are transitory and generally mild to moderate in severity [34]. The pigmented portion of iris also gets heated up during resistance to the flow of the current which can induce denaturation of lenticular proteins and opacification [35]. The lightning strike leads to both anterior and posterior coarse subcapsular opacities. These opacities can often impair vision significantly and necessitate surgical removal [33]. The cataract in our patients was not visually significant, and we proceeded with observation. Similarly, neural injury can occur by several mechanisms. Myelin injury occurs due to resistance for the current flow, coagulation factor abnormalities, and consequential damage to the vascular supply [36].

## 4. Conclusion

The unpredictability of the flow of electric current through a human body results in different modalities of presentations of ocular injury. OCT, a non-invasive technology, is particularly useful in the evaluation of lighting induced maculopathy as well as its evolution through the course of time. Though observation alone may suffice, visual prognosis in lightning induced maculopathy depends upon the degree of involvement of ocular structures. The presence of irreversible retinal damage in terms of retinal atrophy as well as optic nerve damage are the major determinant factors for poor visual outcome in the absence of significant anterior segment pathology. This report signifies the importance of long term follow-up post lightning injury. Though no definite conclusions can be made, we believe that the findings from these two cases will contribute to the pool of knowledge in understanding the evolution of lightning maculopathy.
